# Envenomation with Snake Venoms as a Cause of Death: A Forensic Investigation of the Decomposition Stages and the Impact on Differential Succession Pattern of Carcass-Attracted Coleopteran Beetles

**DOI:** 10.3390/insects15110902

**Published:** 2024-11-18

**Authors:** Abdelwahab Khalil, Abeer M. Salem, El-Sayed H. Shaurub, Ashraf M. Ahmed, Areej A. Al-Khalaf, Mahmoud M. Zidan

**Affiliations:** 1 Entomology Division, Zoology Department, Faculty of Science, Beni-Suef University, Beni-Suef 62521, Egypt; 2 Department of Biotechnology, Faculty of Science, Cairo University, Cairo 12613, Egypt; mabeer@sci.cu.edu.eg; 3 Department of Entomology, Faculty of Science, Cairo University, Cairo 12613, Egypt; sayedshaurub@yahoo.com; 4 Zoology Department, Faculty of Science, King Saud University, Riyadh 11481, Saudi Arabia; aalii@ksu.edu.sa; 5 Biology Department, College of Science, Princes Nourah bint Abdulrahman University, Riyadh 11671, Saudi Arabia; aaalkhalaf@pnu.edu.sa; 6 Zoology & Entomology Department, Faculty of Sciences, Al-Azhar University, Cairo 11884, Egypt; mahmoud.zidan@azhar.edu.eg

**Keywords:** carcasses, venoms, snakes, forensic, necrophilous beetles, decomposition, succession

## Abstract

Background: Coleoptera is the second-most significant insect group associated with decomposing carcasses, yet its role in the decomposition process and postmortem colonization following envenomation is poorly understood. Purpose of the Study: This study aimed to investigate the effects of the venoms from *Cerastes cerastes* and *Naja haje* on the decomposition of rabbit carcasses while evaluating the main beetle taxa attracted to these decaying remains. Methods: Three groups of rabbits, each with five individuals, were utilized. The first group was injected with the venom of *Cerastes cerastes*, the second with *Naja haje* venom, and the control group received 0.85% physiological saline before euthanasia with CO_2_. Results: Four decomposition stages (fresh, bloating, decay, and dry) with durations varying based on venom type and carcass condition were observed. A total of 647 individual beetles of six species (*Necrobia rufipes*, *Attagenus* sp., *Dermestes frischii*, *D. maculatus*, *Bledius* sp., and *Apentanodes* sp.) belonging to four families (Cleridae, Dermestidae, Staphylinidae, and Tenebrionidae) were collected and identified. *D. maculatus* was the most abundant species. Fewer beetles were attracted to carcasses envenomed with *N. haje* compared to the other groups. Conclusions: Envenomation by snake venom influences the attraction and succession rate of necrophilous coleopterans to carcasses, which is important for forensic investigations.

## Simple Summary

This study investigates the impact of envenomation with snake venom on the decomposition of rabbit carcasses and the subsequent attraction of necrophilous coleopteran beetles, a crucial factor in forensic entomology. The research aimed to assess how venoms from the Egyptian cobra (*Naja haje*) and the horned viper (*Cerastes cerastes*) influence decomposition stages and beetle colonization patterns. Results indicated that snake envenomation accelerated the decomposition process, particularly shortening the fresh and bloating stages, and reduced the number of beetles attracted to envenomed carcasses compared to controls. Notably, fewer beetles were associated with *N. haje* envenomed carcasses. The findings highlight the significant role of snake venom in altering carcass decomposition and insect succession, providing valuable insights for forensic investigations. Understanding these relationships can enhance the accuracy of estimating the postmortem interval in legal cases, ultimately contributing to more effective crime scene analyses and improving public safety through more effective forensic practices.

## 1. Introduction

Insects and other arthropods are valuable resources in legal investigations [1]. By studying the insects found on dead bodies, investigators can determine the minimum postmortem interval, PMImin, for a particular species within a specific biogeographical region [2,3]. Various factors, such as the type and condition of the remains, temperature, humidity, and the biogeoclimatic zone, influence the composition of carrion communities. These factors also affect the seasonal availability of insects, the succession of species, and the pace of decomposition [4,5].

The largest insect order is *Coleoptera*, accounting for nearly a third of all known insect species [6]. Necrophilous beetles occupy a diverse ecological niche within the carrion ecosystem, providing a wide range of potential evidence sources for medico-legal investigations [7]. These beetles are crucial not only in assessing the damage and alterations to the posture of corpses but also in estimating the minimum postmortem interval (PMImin) of skeletal remains that are dry and in the final stages of decay [8]. The most forensically significant beetle families include *Dermestidae*, *Scarabaeidae*, Staphylinidae, *Cleridae*, and *Histeridae*, whose existence may vary by region [9]. While most research on insect development related to carrion has focused on flies, much less attention has been given to necrophilous beetles [10].

Envenomation by poisonous animals and arthropods is one of the leading causes of death in humans [11,12,13,14]. Snake, scorpion, and spider venoms affect millions of people worldwide, particularly in Africa, Asia, the Middle East, and Latin America [15,16,17,18,19,20,21]. In Egypt, venomous animals cause over 10,000 bites and approximately 200 deaths annually [22]. In addition to their lethality, snake venom envenomation causes significant tissue damage, leading to tissue loss and dysfunction [23].

Deadly snakebites are difficult to identify by internal anatomy at autopsy in forensic investigations [14,24], and the clinical presentation is often the only reliable method for determining the type of snake involved [25]. While several studies have explored the toxic effects of snake venoms [23,26,27,28,29,30,31,32,33], little is known about how these venoms impact the decomposition process and the succession pattern of necrophilous beetles. Therefore, investigating how envenomation influences both decomposition and insect succession on carcasses could provide valuable physical evidence in criminal investigations.

The aim of this study was to examine the effects of envenomation with the venoms of *Cerastes cerastes* and *Naja haje* on the decomposition process of rabbit carcasses and to evaluate the main beetle taxa associated with decaying carcasses quantitatively and qualitatively.

## 2. Materials and Methods

### 2.1. Site of Study and Meteorological Measurements

**Figure 1:**
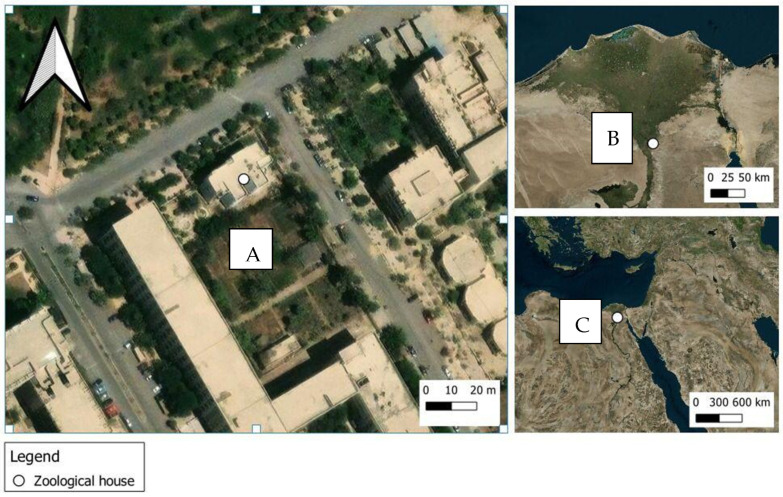
Maps showing the location of the study site. (**A**): College of Science garden, Al-Azhar University Nasr City, Cairo, where the study was carried out. (**B**): Cairo. (**C**): Egypt.

This research was conducted from the 2nd to the 17th of August 2022 at the College of Science Garden, Al-Azhar University, Nasr City, Cairo, Egypt (30.060055, 31.314697) (Figure 1). Relative humidity, ambient temperature (°C), wind speed, carcass temperatures, and soil temperature were measured daily, following the methodology detailed in Khalil et al. (2023) [34].

### 2.2. Animals and Venoms

*Oryctolagus cuniculus domesticus*, a species of European domestic rabbit (Lagomorpha, Leporidae), weighing approximately 2.5 kg each, were utilized as the animal model for this investigation, according to earlier studies [35,36]. The rabbits were obtained from the Egyptian Holding Company of Biological Products and Vaccines (VACSERA) Animal House (https://www.vacsera.com/#home, accessed on 14 September 2024). The animals were handled carefully to prevent injuries, as external wounds could attract flies and alter the patterns of succession. The venoms used in this study were obtained from two native snake species: the Egyptian cobra (*Naja haje*), one of the deadliest snakes in Africa and the world, and the horned viper (*Cerastes cerastes*), which inhabits desert regions of North Africa, including Egypt. The venom was generously donated by the Africano Tolba Zoo Company, located in Giza, Egypt. According to Khalil et al. (2023) [34], the venoms were dissolved in 0.85% physiological saline (pH 7.2) to achieve a final aliquot stock concentration of 1 mg/mL (*w*/*v*) prior to use in experiments. This preparation followed the methodology described by Broad et al. (1979) [37].Before being used in experiments, and as stated by Khalil et al., (2023) [34], venoms were dissolved in 0.85% physiological saline (pH 7.2) to obtain a final aliquot stock concentration of 1 mg/mL (*w*/*v*) in accordance with Broad et al., (1979) [37].

### 2.3. In Vivo Determination of LD_50_ of Venoms

The LD_50_ of *N. haje* and *C. cerastes* venoms was evaluated in accordance with the 2007 World Health Organization (WHO) guidelines, as well as the methods outlined by Khalil et al. (2023) [34].

### 2.4. Envenomation of Rabbits

Three groups of rabbits, each consisting of five rabbits (*n* = 5), were used in this study. The lethal doses (LD_95_) of the venoms studied were calculated as 12.8 and 62.0 μg/g, respectively, as detailed in Khalil et al. (2023) [34]. These doses were intravenously injected into the first and second groups, while the third group served as a control and was injected with 0.85% physiological saline. After being injected with either *Naja haje* or *Cerastes cerastes*, the rabbits were left in their cages until death occurred, which was observed at 10 and 20 min post-injection, respectively. The control group’s rabbits were euthanized with CO_2_, following the protocols outlined by Conlee et al. (2005) [38]. Upon confirmed death, the rabbit carcasses were moved to the experimental site as soon as possible, within a maximum timeframe of one hour. The experimental design and settings were adapted from our previous work [34].

### 2.5. Collection and Identification of Beetles

During the sampling period, adult beetles were collected daily and quickly preserved in vials containing 70% ethanol. Each vial was labeled with the date and corpse number. The collected beetles were then identified and categorized into families and species using dichotomous keys [39,40,41].

### 2.6. Statistical Analyses

Due to the non-normal distribution of most datasets, significant variance differences between treatment groups, and small sample sizes, Kruskal–Wallis H-tests were employed to assess statistical differences in (1) the duration of decomposition stages among the control and envenomed rabbit carcasses, (2) the number of beetles attracted to rabbit carcasses each day across the treatment groups (control, *N. haje*, and *C. cerastes*), (3) the number of coleopteran families recorded among the different treatment groups, and (4) the number of beetles observed at each decomposition stage (both at the aggregate and individual family levels) across the treatment groups. Dunn’s test was subsequently used for pairwise multiple comparisons. Student’s *t*-test was applied to compare minimum and maximum values for each meteorological parameter. All statistical analyses were conducted using IBM SPSS Statistics Version 23 (IBM Corp., Armonk, NY, USA) with a significance level of *p* < 0.05.

## 3. Results

### 3.1. Meteorological Data

Table 1 shows that there was a significant difference (two-sample *t*-test, df = 8, *p* < 0.05) between the maximal and minimal temperatures of the surrounding environment, which were 36.80 ± 0.39 °C and 24.93 ± 0.18 °C, respectively. Additionally, there was a significant difference (*p* < 0.05, two-sample *t*-test, df = 8) between the maximal and minimal atmospheric relative humidity levels, recorded as 85.40 ± 0.42% and 30.06 ± 0.06%, respectively. The mean soil temperature and relative humidity throughout the experimental period were 42.93 ± 0.34 °C and 59.13 ± 0.43%, respectively. The mean wind speed was 15.26 ± 0.33 km/h. Regarding the mean temperature of the carcasses, there was no significant difference (*p* > 0.05, Two-Sample *t*-test, df = 8) between the carcasses envenomed with *N. haje* venom (15.63 ± 1.71 °C) or *C. cerastes* venom (14.93 ± 1.92 °C) when compared to the control carcasses (15.87 ± 1.59 °C).

### 3.2. Decomposition Stages

Table 2 shows the decomposition stages in non-envenomed carcasses (control) and envenomed carcasses, viz. the fresh stage, bloating stage, decay stage, and dry stage. The duration of decomposition stages varied significantly among the control and envenomed rabbit carcasses. In the fresh stage, control carcasses exhibited a significantly longer duration (2 ± 0.14 days) compared to both *N. haje*-envenomed(1 ± 0.16 days) and *C. cerastes*-envenomed (1 ± 0.10 days)carcasses (H = 9.239, df = 2, *p* = 0.01).The carcasses appeared the same as before death(soft and no bad odor). During the bloating stage, significant differences were observed among treatments (H = 10.102, df = 2, *p* = 0.006). This stage lasted for 5 days (from day 2 to day 6 postmortem) and 3 days (from day 2 to day 4 postmortem) in the carcasses envenomed with *N. haje* and *C. cerastes* venoms, respectively, compared to 5 days (from day 3 to day 7 postmortem) in the control carcasses. In the bloating stage, the abdomen begins to swell, with the appearance of a slight putrefy odor. The decay stage showed no significant differences in duration among the three groups (H = 0.049, df = 2, *p* = 0.976), with control, *N. haje*, and *C. cerastes*-envenomed carcasses taking approximately 6 days to complete this stage (6 ± 0.08, 6 ± 0.21, and 5 ± 0.20 days, respectively). This stage started from day 7 to day 12 in *N. haje*-envenomed carcasses, from day 5 to day 10 in *C. cerastes*-envenomed carcasses, and from day 8 to day 13 in the control carcasses postmortem. In the decay stage, masses of coleopteran larvae fed on soft tissues and most of the body fluids had leaked out, with a strong putrid odor of decay. The dry stage, which is the final stage, started from day 13 and 11 postmortem in the carcasses envenomed with *N. haje* and *C. cerastes* venoms, respectively, compared to day 14 postmortem in the control carcasses. Significant differences were observed among the control and envenomed carcasses (H = 12.299, df = 2, *p* = 0.002). In this stage, the carcasses became very hard/rigid and all the soft tissue and flesh had been consumed, with significant decline in the putrid odor. The carcasses consisted only of hard tissues: bones, skin, and hair. These data may indicate that snake venoms had shortened the fresh, bloating, and decay stages compared to the controls. Also, it appears that *C. cerastes*-envenomed carcasses showed the shortest decomposition stages, and thus, reached the dry stages faster (day 11 postmortem) compared with both *N. haje*-envenomed and control ones (day 13 and 14 postmortem, respectively).

### 3.3. Differential Succession of Coleopterans

Table 3 shows that the collected beetles belonged to six species and four families. The family Dermestidae, with three species identified, accounted for 82.23% of all the beetles collected, making it the most abundant group. These results indicate that dermestids were the predominant visitors to the carcasses throughout the experimental period. Among the dermestids, *D. maculatus* was the most abundant species, accounting for 65.04% of all collected dermestids and 53.48% of the total number of all collected beetles. Following *D. maculatus*, *D. frischii* and *Attagenus* sp. were the second and third most predominant dermestids, respectively. They represented 22.37% and 12.59% of the total dermestids, and 18.39% and 10.36% of the total beetles, respectively. The Tenebrionidae family was the second-most abundant family (10.05% of the collected beetles) and was represented by only one species, *Apentanodes* sp. The Cleridae and Staphylinidae families were the third- and fourth-most abundant, accounting for 6.03% and 1.70% of the total collected beetles, respectively. Each of these families was represented by a single species: *N. rufipes* for Cleridae and *Bledius* sp. for Staphylinidae. All collected clerid and dermestid beetles from both envenomed and non-envenomed (control) carcasses were recorded during four stages of decomposition, except for the fresh stage. *Bledius* sp. appeared only in the decay stage of control carcasses and in the bloating, decay, and dry stages of envenomed carcasses. *Apentanodes* sp. was observed in the bloating, decay, and dry stages of both control and *N. haje*-envenomed carcasses, as well as in the bloating and decay stages of *C. cerastes*-envenomed carcasses. Thus, it appears that there was no relationship between specific beetle species and the stages of decomposition following envenomation.

### 3.4. Abundance of Coleopterans Attracted to Carcasses

**Figure 2:**
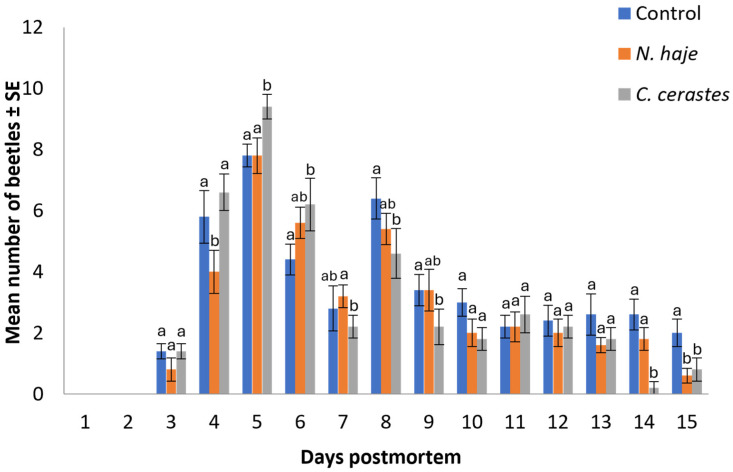
Mean number of beetles attracted to rabbit carcasses upon envenomation with snake venoms throughout the experimental period (15 days). Error bars represent the SE of means of 5 replicates (*n* = 5), using 5 different individual rabbits in each treatment. For each day, bars followed by different letters denote significant differences in the mean number of beetles (Kruskal–Wallis H-test, Dunn’s test, *p* < 0.05).

A total of 647 beetles were collected from all experimental carcasses in this study. The mean number of beetles attracted to envenomed rabbit carcasses over the experimental period (15 days) is shown in Figure 2. The pattern of beetle collection follows an inverted V-shape, with mean numbers of 46.80 ± 6.88, 40.40 ± 5.98, and 42.00 ± 6.05 beetles (n = 5) recorded on the control, *Naja haje* -envenomed, and *Cerastes cerastes*-envenomed carcasses, respectively. It is noticeable that envenomation with snake venoms significantly decreased the mean number of beetles attracted to carcasses compared to the control, particularly in those envenomed with the more lethal *N. haje* venom (H = 1.40–11.08, df = 2, *p* = 0.004–0.497). Beetles began appearing on both control and envenomed carcasses starting on day 3 postmortem. The maximum abundance of beetles was recorded on day 5 postmortem in the control (7.80 ± 0.37), *N. haje*-envenomed carcasses (7.80 ± 0.58), and *C. cerastes*-envenomed carcasses (9.40 ± 0.40). A significant decline in beetle numbers was observed on days 14 and 15 postmortem in *C. cerastes*-envenomed (0.20 ± 0.10) and *N. haje*-envenomed carcasses (0.60 ± 0.25), respectively.

**Figure 3:**
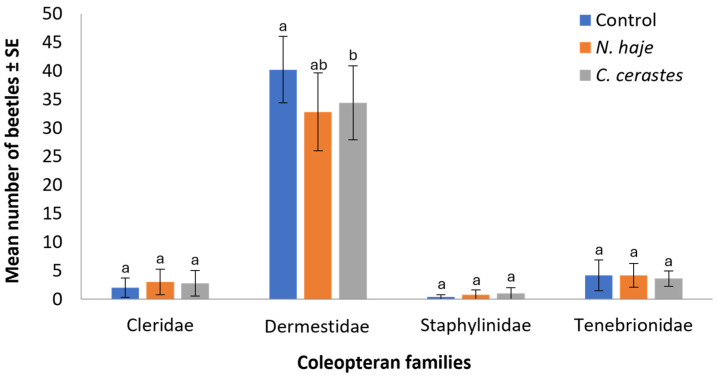
Mean number of recorded coleopteran families upon envenomation with snake venoms throughout the experimental period (15 days). Error bars represent the SE of means of 5 replicates (*n* = 5), using 5 different individual rabbits in each treatment. For each family, bars followed by different letters denote significant differences in the mean number of beetles (Kruskal–Wallis H-test, Dunn’s test, *p* < 0.05).

The effect of venom type on the abundance of coleopteran families during the experimental period is shown in Figure 3. The abundance of beetles collected from rabbit carcasses varied across treatment groups and families. Dermestidae was the most abundant family across all families, with the control group showing the highest mean number (40.2 ± 5.83 beetles), followed by *C. cerastes* (34.4 ± 6.5) and *N. haje* (32.8 ± 6.83) treatments (H = 9.93, df = 2, *p* = 0.007). The remaining three families (Cleridae, Staphylinidae, and Tenebrionidae) showed considerably lower abundance, with mean counts typically below five beetles across all treatment groups. For these families, no significant differences were observed between control and venom-treated carcasses (H = 2.21–2.79, df = 2, *p* = 0.261–0.330). Specifically, Cleridae showed mean counts of 2.0 ± 1.69, 3.0 ± 2.23, and 2.8 ± 2.22 beetles for control, *N. haje*, and *C. cerastes* treatments, respectively. Staphylinidae were the least abundant, with means below one beetle per treatment, while Tenebrionidae showed slightly higher numbers (4.2 ± 2.71, 4.2 ± 2.10, and 3.6 ± 1.33 for control, *N. haje*, and *C. cerastes* treatments, respectively). These results suggest that, although envenomation may exert a slight influence on the abundance of Dermestidae beetles, it does not significantly impact the colonization patterns of other beetle families during the decomposition process.

**Figure 4:**
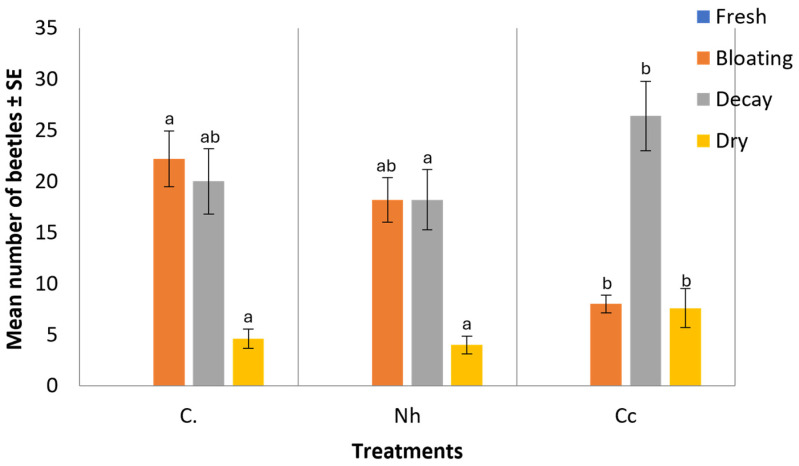
Mean number of recorded beetles in each decomposition stage. Error bars represent the SE of means of 5 replicates (*n* = 5), using 5 different individual rabbits in each treatment. C: control carcasses; Nh: *N. hajie*-envenomed carcasses; Cc: *C. cerastes*-envenomed carcasses. Different letters above bars indicate significant differences in beetle abundance between treatment groups within each decomposition stage (Kruskal–Wallis test followed by Dunn’s post hoc test, *p* < 0.05).

Figure 4 shows significant differences that were reported for the abundance of beetles observed at each decomposition stage (H = 9.96–12.34, df = 2, *p* = 0.002–0.007). During the fresh stage of both non-envenomed and envenomed rabbit carcasses, no beetles were observed. However, during the bloating stage, an average of 18.20 ± 2.17 beetles was collected from *N. haje*-envenomed carcasses, and 8.0 ± 0.85 from *C. cerastes*-envenomed carcasses, compared to a mean of 22.20 ± 2.73 beetles collected in the control group. In the decayed stage, the mean number of beetles recorded was 18.20 ± 2.95 for *N. haje*-envenomed carcasses, 26.40 ± 3.39 for *C. cerastes*-envenomed carcasses, and 20.0 ± 3.20 in the control carcasses. Finally, during the dry stage, 4.0 ± 0.87 beetles were recorded in *N. haje*-envenomed carcasses, 7.60 ± 1.91 in *C. cerastes*-envenomed carcasses, and 4.60 ± 0.96 beetles in the control group.

**Figure 5:**
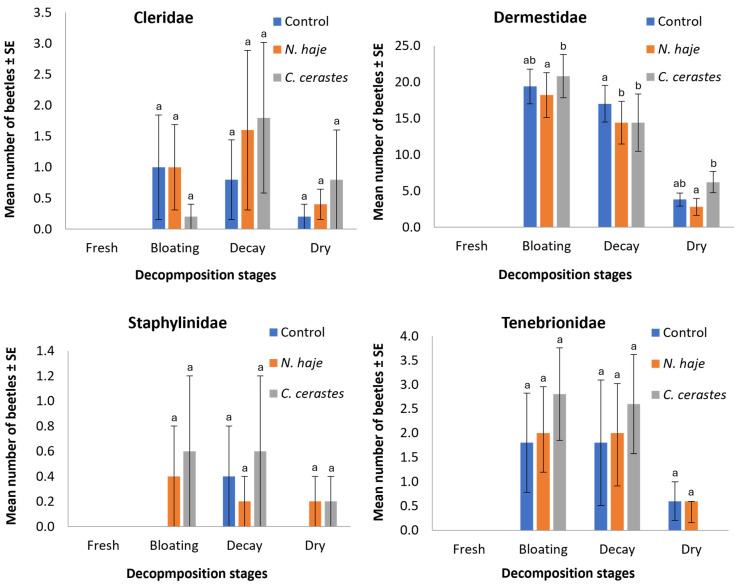
Mean number of recorded coleopteran families in each decomposition stage. Error bars represent the SE of means of 5 replicates (*n* = 5), using 5 different individual rabbits in each treatment. The control group consisted of rabbits injected with 0.85% physiological saline and euthanized with CO_2_, serving as a unified reference point across all coleopteran families. For each decomposition stage, bars followed by different letters denote significant differences in the mean number of beetles (Kruskal–Wallis H-test, Dunn’s test, *p* < 0.05).

Figure 5 illustrates the effects of envenomation on the mean number of individuals within each family throughout the four stages of decomposition. All known beetle families were present in varying amounts during carcass decomposition. In the fresh stage, no beetles were recorded. The mean number of beetles within Cleridae in carcasses envenomed with *N. haje* and *C. cerastes* was 1.0 ± 0.69 and 0.2 ± 0.2, 1.6 ± 1.29 and 1.8 ± 1.22, and 0.4 ± 0.25 and 0.8 ± 0.8 during the bloating, decay, and dry stages, respectively, compared to 1.0 ± 0.85, 0.8 ± 0.65, and 0.2 ± 0.2 in the non-envenomed carcasses for the same stages. The highest number of beetles was recorded within Dermestidae, with the mean number recorded in envenomed carcasses being 18.2 ± 3.11 and 20.8 ± 2.98, 14.4 ± 2.96 and 21.6 ± 3.95, and 2.8 ± 1.16 and 6.2 ± 1.47 during the bloating, decay, and dry stages, respectively, compared to 19.4 ± 2.39, 17.0 ± 2.53, and 3.8 ± 0.91 in the non-envenomed carcasses for the same stages. For Staphylinidae, the mean number recorded in the envenomed carcasses was 0.4 ± 0.4 and 0.6 ± 0.6, 0.2 ± 0.2 and 0.6 ± 0.6, and 0.2 ± 0.2 and 0.2 ± 0.2 in the bloating, decay, and dry stages, respectively, compared to 0.0 ± 0.0, 0.4 ± 0.4, and 0.0 ± 0.0 in the non-envenomed carcasses. Regarding Tenebrionidae, the mean number recorded in envenomed carcasses was 2.0 ± 0.81 and 2.8 ± 0.96, 2.0 ± 1.09 and 2.6 ± 1.02, and 0.6 ± 0.45 and 0.0 ± 0.0 during the bloating, decay, and dry stages, respectively, compared to 1.8 ± 1.02, 1.8 ± 1.29, and 0.6 ± 0.4 in the non-envenomed carcasses for the same stages. Dermestidae showed the most pronounced differences among groups and was the only family exhibiting statistically significant variations (H = 9.01–11.79, df = 2, *p* = 0.003–0.011). The remaining three families showed no significant differences across treatments in any decomposition stage (H = 1.07–5.38, df = 2, *p* = 0.068–0.584).

## 4. Discussion

This study is a preliminary step towards enriching our understanding of how the presence of animal venoms in carcasses can affect both the rate of decomposition and the succession pattern of forensically important coleopterans. Although the biogeoclimatic zone is the most significant factor influencing carcass decomposition [42,43,44,45], extrinsic factors, including the cause of death (e.g., drugs, toxins, and poisons), also play a role in both decomposition and insect succession patterns [21,46,47,48,49]. In this context, envenomation by animal and arthropod venoms is considered one of the leading causes of human mortality [13,17,19,34,50], contributing to fatalities worldwide [1,14,25] and in Egypt [21]. However, in Egypt, little attention has been given to studying the effects of envenomation by scorpion [50] and snake [34] venoms on the decomposition process and the succession patterns of major necrophilous beetles and Diptera, respectively, using rabbits as animal models. To the best of our knowledge, this is the first work to statistically and qualitatively demonstrate the effect of snake venom envenomation on the necrophilous beetle’s succession pattern and the decomposition process of rabbit carcasses in Egypt.

August is the hottest and driest month in Cairo; characterized by stable climatic conditions, with an atmospheric temperature of 24.93–36.80 °C, an atmospheric relative humidity of 30.06–85.40%, an atmospheric wind velocity of 15.26 km/h, a soil temperature of 42.93 °C, and a soil humidity of 59.13%. The results suggest that there was no external influence on the postmortem succession of coleopteran species in this study. The environmental conditions remained stable throughout the 15-day duration. Therefore, it is hypothesized that ante-mortem envenomation with snake venom, as the primary cause of death, may explain the relative decrease in the number of beetles colonizing the envenomed rabbit carcasses (abundance) compared to those inhabiting the un-envenomed rabbit carcasses. In this context, *N. haje* venom was more toxic than *C. cerastes* venom, with LD_50_ values of 12.8 μg/g and 62.0 μg/g, respectively. Consequently, coleopteran abundance was lower in carcasses envenomed with *N. haje* venom than in those envenomed with *C. cerastes* venom.

Regarding meteorological data, the temperature of envenomed carcasses was consistently lower than that of the control carcasses, despite the difference between the two being insignificant. Similarly, Abdou and Ibrahim (2015) [23] recorded that shortly after death, the temperature of rats envenomed with *N. haje* venom decreased to 33 °C, compared to 36 °C in the control rats. According to Abdou and Ibrahim (2015) [23], postmortem changes and metabolic deterioration may be responsible for the lower temperature caused by envenomation. Additionally, the temperature of the rabbit carcasses was approximately three times lower than that of the soil, while the soil temperature was about twice as high as the air temperature.

Previous studies have classified the decomposition process into 4–5 stages [50,51,52,53,54,55,56], depending on various factors such as habitat, vegetation, soil type, seasonal temperature, gut microbiome, size of the corpse, cause of death, burial conditions, and the presence or absence of clothing [43,44,45,57,58,59]. We also documented four decomposition stages (fresh, bloating decay, and dry) in this study and our earlier work [34], with decomposition durations that differed depending on the type of venom and state of decomposition in both *N. haje*- and *C. cerastes*-envenomed carcasses. The fresh stage was shorter by one day compared to that of the non-envenomed carcasses (control). The bloating stage in *C. cerastes*-envenomed carcasses was two days shorter than in the non-envenomed carcasses. Thus, we concluded that envenomation accelerated the decomposition process during the early stages. Conversely, compared to the control, the dry stage in *N. haje*- and *C. cerastes*-envenomed carcasses significantly increased by one and three days, respectively (H = 12.299, df = 2, *p* = 0.002). Abd El-Aziz et al. (2022) [50] reported that the decomposition rate for each stage in rabbit carcasses envenomed with scorpion venom was shorter than that of the control carcasses. Furthermore, the decomposition of envenomed rabbits was accelerated by intoxication with additional substances such as heroin [60] and alcohol [61].

The venom of *N. haje* contains several enzymatic and non-enzymatic proteins, along with other substances that work together as neuro-cardiotoxins [29,62]. When bitten by *N. haje*, studies show severe damage to the liver, kidney, heart, brain, and lungs [23,26,29,31,33]. There is also evidence of blood and biochemical changes after envenomation, like polycythemia [23,28,30], increased C-reactive protein levels [23,63], decreased ATP in skeletal and cardiac muscles [23,64,65,66], and proteolytic effects [65]. On the other hand, the venom of *Cerastes* is packed with harmful proteins like metalloproteinases, disintegrins, procoagulants, cerastocytin, phospholipase, and C-type lectin-like proteins [67,68]. These work together to mess with the body’s ability to clot blood, causing persistent bleeding [69,70]. *C. cerastes* venom has also been reported to have significant cytotoxic effects [69,70]. Consequently, the different toxicological and pharmacological effects of *N. haje* and *C. cerastes* venom seem to explain why envenomed rabbit carcasses decomposed faster in this study.

When the venoms of *N. haje* and *C. cerastes* were applied to rabbit carcasses, the sequence of recorded coleopteran families and species revealed that the most common visitors to the carcasses were members of the Dermestidae family. Among the dermestids, *D. maculatus* was the most abundant species, followed by *D. frischii* and *Attagenus* sp. The Tenebrionidae family was the second-most abundant coleopteran family, followed by Cleridae and Staphylinidae. Both Cleridae and Dermestidae were recorded during four decomposition stages, except for the fresh stage. *Bledius* sp. (Staphylinidae) appeared only during the decay stage of control beetles and in the bloating, decay, and dry stages of envenomed carcasses. *Apetanodes* sp. (Tenebrionidae) was observed in the bloating, decay, and dry stages of both control and *N. haje*-envenomed carcasses, as well as in the bloating and decay stages of *C. cerastes*-envenomed carcasses. Therefore, there does not seem to be any connection between certain beetle species and the phases of decomposition that occurred after envenomation. In agreement with our results, Mashaly (2017) [71] showed that the predominant coleopteran species on exposed rabbit carcasses in Saudi Arabia belonged to the families Dermestidae and Histeridae. They reported no relationship between the occurrence of a single species and a particular stage of decomposition. Sonker et al. (2019) [72] found that the beetles arriving on exposed pig carcasses in India primarily belonged to the families Histeridae, Dermestidae, and Cleridae. Al-Dakhil and Alharbi (2020) [73] identified four coleopteran families on exposed rabbit carcasses in Saudi Arabia: Histeridae (*Euspilotus modestus* Erichson), Dermestidae (*Dermestes maculates*), Cleridae (*Necrobiarufipes*), and Scarabaeidae (unidentified). Among these species, *D. maculatus* was the most abundant. In the present study, *Dermestes* sp. were the dominant species collected from rabbit carcasses during the bloating, decay, and dry stages. Early and Goff (1986) [74] and Mabika et al. (2014) [75] collected *Dermestes* sp. only during the bloating and decay stages, respectively. According to VanLaerhoven and Anderson (1999) [76], the early presence of these beetles in the decay process might be due to their peak seasonal appearance rather than the decomposition state of the carcass.

## 5. Conclusions

The effect of envenomation with the venoms of *N. haje* and *C. cerastes* snakes on the decomposition and succession pattern of forensically significant coleopteran taxa, using rabbit carcass as a model, was examined in this study as a cause of death. Therefore, our results can be of forensic importance. In our earlier investigation, we demonstrated [34] that envenomation shortened the total length of the four decomposition stages when compared to the control group. The current study showed no relationship between specific beetle species and the stages of decomposition following envenomation. Moreover, envenomation also reduced the abundance of carcass-attracted beetles, with the impact of the highly lethal *N. haje* venom being relatively greater than that of the less lethal *C. cerastes* venom.

The Dermestidae family was the most abundant coleopteran family attracted to envenomed carcasses, followed by the Tenebrionidae, Cleridae, and Staphylinidae families. Among the identified species, *Dermestes maculatus* was the most abundant. These results imply that differences in the type of venom causing death may lead to variations in the quantity and composition of beetles drawn to carcasses.

Future research should clarify the impact of snake venom envenomation on the growth of beetles associated with carcasses. Toxicological and cytotoxic analyses should be employed to identify venom residues in the tissues of both the related insects and the carcasses. This approach could enhance our understanding of how envenomation affects the decomposition of carcasses and the succession patterns of insects in specific geographic areas. Such knowledge could be valuable in forensic investigations, particularly in using insect species to estimate the postmortem interval.

## Figures and Tables

**Table 1:** Mean values of meteorological parameters of the surrounding environment and carcasses in the experimental site over the experimental period (15 days).

Atmospheric Temperature (°C)		Atmospheric Relative Humidity (%)		Soil Temperature (°C)	Soil Humidity (%)	Wind Speed (km/h)	Carcasses Temperature (°C)		
Maximum	Minimum	Maximum	Minimum				Control	*N. haje*	*C. cerastes*
36.80 ± 0.39	24.93 ± 0.18	85.40 ± 0.42	30.06 ± 0.06	42.93 ± 0.34	59.13 ± 0.43	15.26 ±0.33	15.87 ± 1.59	15.63 ± 1.71	14.93 ± 1.92

**Table 2:** Decomposition stages of rabbit carcasses envenomed with *N. haje* and *C. cerastes* venoms over 15 days. Duration of each stage expressed as mean ± SE.

Treatments	Days Postmortem ± SE														
	1	2	3	4	5	6	7	8	9	10	11	12	13	14	15
Control	2 ± 0.14a *		5 ± 0.16a					6 ± 0.08a						2 ± 0.17a	
*N. haje*	1 ± 0.16b	5 ± 0.07a					6 ± 0.21a						3 ± 0.16ab		
*C. cerastes*	1 ± 0.10b	3 ± 0.15b			6 ± 013a						5 ± 0.20b				
	Key:	Fresh			Bloated			Decayed			Dried				
						

* Different letters vertically within the same color denote significant differences (Kruskal–Wallis H-test, Dunn’s test, *p* < 0.05).

**Table 3:** Recorded coleopteran families and species at different stages of rabbit carcasses decomposition upon envenomation with *N. haje* and *C. cerastes* venoms.

Coleopteran Families (Number/Five Carcasses)	Beetle Species (Number/Five Carcasses)	Control				*N. hajie*				*C. cerastes*			
		Fr	Bl	De	Dr	Fr	Bl	De	Dr	Fr	Bl	De	Dr
Cleridae (39)	*Necrobia rufipes* (Fabricius, 1781) (39)	−	+	+	+	−	+	+	+	−	+	+	+
Dermestidae (532)	*Attagenus* sp. (67)	−	+	+	+	−	+	+	+	−	+	+	+
	*Dermestes* *Frischii* Kugelann, 1792 (119)	−	+	+	+	−	+	+	+	−	+	+	+
	*D. maculatus* De Geer, 1774 (346)	−	+	+	+	−	+	+	+	−	+	+	+
Staphylinidae (11)	*Bledius* sp. (11)	−	−	+	−	−	+	+	+	−	+	+	+
Tenebrionidae (65)	*Apentanodes* sp. (65)	−	+	+	+	−	+	+	+	−	+	+	−

Fr: fresh stage, Bl: bloating stage, De: decayed stage, Dr: dry stage. Positive sign (+) indicates recorded beetles.

## Data Availability

All data generated or analyzed during this study are included in the present article.

## References

[1] Byrd J.H., Tomberlin J.K. (2019). Forensic Entomology: The Utility of Arthropods in Legal Investigations.

[2] Watson E.J., Carlton C.E. (2003). Spring succession of necrophilous insectson wildlife carcasses in Louisiana. J. Med. Entomol..

[3] Matuszewski S., Bajerlein D., Konwerski S., Szpila K. (2010). Insect succession and carrion decomposition in selected forests of CentralEurope. Part 2: Composition and residency patterns of carrion fauna. Forensic Sci. Int..

[4] Mann R.W., Bass W.M., Meadows L. (1990). Time since death and decomposition of the human body: Variables and observations incase and experimental field studies. J. Forensic Sci..

[5] Anderson G.S., Byrd J.H., Castner J.L. (2010). Factors that influence insect succession on carrion. Forensic Entomology: The Utility of Arthropods in Legal Investigations.

[6] Byrd J.H., Castner J.L. (2010). Forensic Entomology: The Utility of Arthropods in Legal Investigations.

[7] Schoenly K.G., Haskell N.H., Hall R.D., Gbur J.R. (2007). Comparative performance and complementarity of four sampling methods and arthropod preference tests from human and porcine remains at the Forensic Anthropology Center in Knoxville, Tennessee. J. Med. Entomol..

[8] Kulshrestha P., Satpathy D.K. (2001). Use of beetles in forensic entomology. Forensic Sci. Int..

[9] Hart A.J., Whitaker A.P. (2006). Forensic entomology. Antenna.

[10] Guo Y.D., Cai J.F., Xiong F., Wang H.J., Wen J.F., Li J.B., Chen Y.Q. (2012). The utility of mitochondrial DNA fragments forgeneticidentificationofforensicallyimportantsarcophagidflies(Diptera:Sarcophagidae)inChina. Trop. Biomed..

[11] Biery T.L. (1977). Venomous Arthropod Handbook: Envenomation Symptoms/Treatment, Identification, Biology and Control.

[12] Edstrom A. (1992). Venomous and Poisonous Animals.

[13] Forrester J.A., Weiser T.G., Forrester J.D. (2018). An Update on Fatalities Due to Venomous and Non venomous Animals in the United States (2008–2015). Wilderness Environ. Med..

[14] Forrester J.D., Forrester J.A., Tennakoon L., Staudenmayer K. (2018). Mortality, hospital admission, and health care cost due to injury from venomous and non-venomous animal encounters in theUSA: 5-year analysis of the National Emergency Department Sample. Trauma. Surg. Acute Care Open.

[15] Gutié rez J.M., Theakston R.D., Warrell D.A. (2006). Confronting the neglected problem of snakebite envenoming: The need for a global partnership. PLoS Med..

[16] World Health Organization (WHO) (2007). Rabies and Envenomings: A Neglected Public Health Issue.

[17] Chippaux J.P., Goyffon M. (2008). Epidemiology of scorpionism: A global appraisal. Acta Trop..

[18] Alirol E., Sharma S.K., Bawaskar H.S., Kuch U., Chappuis F. (2010). Snake bite in SouthAsia: Areview. PLoS Negl. Trop. Dis..

[19] Coelho P., Sousa P., Harris D.J., van der Meijden A. (2014). Deep intraspecific divergences in the medically relevant fat-tailed scorpions (Androctonus, Scorpiones). Acta Trop..

[20] Cichutek K., Epstein J., Griffiths E., Hindawi S., Jivapaisarnpong T., Klein H., Minor P., Moftah F., Reddy V., Slamet L. (2017). WHO Expert Committee on Biological Standardization sixty-seventh report. Tech. Rep. Ser. WHO.

[21] Amr Z.S., AbuBaker M.A., Warrell D.A. (2020). Terrestrial venomous snakes and snakebites in the Arab countries of the Middle East. Toxicon.

[22] Kasturiratne A., Wickremasinghe A.R., de Silva N., Gunawardena N.K., Pathmeswaran A., Premaratna R., Savioli L., Lalloo D.G., de Silva H.J. (2008). The global burden of snakebite: A literature analysis and modeling based on regional estimates of envenoming and deaths. PLoS Med..

[23] Abdou R.H., Ibrahim A.E. (2015). Effects of Egyptian cobra (*Naja haje*)venom on postmortem changes and some biochemical parameters in rats. Forensic Sci..

[24] Sant S.M., Purandare N.M. (1972). Autopsy study of cases of snakebite with special reference to the renal lesions. J. Postgrad. Med..

[25] Pathmeswaran A., Kasturiratne A., Fonseka M., Nandasena S., Lalloo D.G., de Silva H.J. (2006). Identifying the biting species in snakebite by clinical features: An epidemiological tool for community surveys. Trans. R. Soc. Trop. Med. Hyg..

[26] Tan N. (1991). The biochemistry of venoms of some venomous snakes of Malaysia: A review. Trop. Biomed..

[27] Omran M., Abdel-Nabi I., El-Naggar M. (1997). Serum biochemical and hormonal parameters as biomarkers for the toxic effects of Egyptian cobra *(Naja haje*) envenomation. J. Nat. Toxins.

[28] Schafer A.I. (2004). Thrombocytosis. N. Engl. J. Med..

[29] Cher C.D., Armugam A., Zhu Y.Z., Jeyaseelan K. (2005). Molecular basis of cardiotoxicity upon cobra envenomation. Cell Mol. Life Sci..

[30] Sejrsen K., Hvelplund T., Nielsen M.O. (2006). Ruminant Physiology: Digestion, Metabolism and Impact of Nutrition on Gene Expression, Immunology and Stress.

[31] Fung S.Y., Tan N.H., Liew S.H., Sim S.M., Aguiyi J.C. (2009). The protective effects of *Mucuna pruriens* seed extract against histopathological changes induced by Malayan cobra (*Naja sputatrix*) venom in rats. Trop. Biomed..

[32] Al-Sadoon M.K., Moneim A.E.A., Diab M., Bauomy A. (2013). Hepatic and renal tissue damages induced by *Cerastes cerastes* gasperetti crude venom. Life Sci. J..

[33] Tohamy A.A., Mohamed A.F., Moneim A.E.A., Diab M.S. (2014). Biological effects of *Naja haje* crude venom on the hepatic and renal tissues of mice. J. King Saud. Univ. Sci..

[34] Khalil A., Zidan M.M.M., Alajmi R., Ahmed A.M. (2023). Impact of Envenomation with Snake venoms on Rabbit Carcass Decomposition and Differential Adult Dipteran Succession Patterns. J. Med. Entomol..

[35] Mapara M., Thomas B.S., Bhat K.M. (2012). Rabbit as an animal model for experimental research. Dent. Res. J..

[36] Mashaly A., Al-Khalifa M., Al-Qahtni A. (2020). *Chrysomya albiceps* Wiedemann (Diptera: Calliphoridae)colonizing poisoned rabbit carcasses. Entomol. Res..

[37] Broad A., Sutherland S., Coulter A.R. (1979). The lethality in mice of dangerous Australian and other snake venom. Toxicon.

[38] Conlee K.M., Stephens M.L., Rowan A.N., King L.A. (2005). Carbondioxide for euthanasia: Concerns regarding pain anddistress, with special reference to mice and rats. Lab. Anim..

[39] Gerstmeier R. (1988). Checkered Beetles. Illustrated Key to the Cleridae and Thanerocleridae of the Western Palaearctic.

[40] Háva J. (2004). World keys to the genera and sub genera of Dermestidae (Coleoptera), with descriptions, nomenclature and distributional records. Acta Musei Nat. Pragae Ser. B Nat. Hist..

[41] Mazur S., Löbl I., Löbl D. (2004). Family Histeridae. Catalogue of Palaearctic Coleoptera: Hydrophiloidea—Staphylinoidea.

[42] Shapiro S.S., Wilk M.B. (1965). An analysis of variance test for normality (complete samples). Biometrika.

[43] Voss S.C., Cook D.F., Dadour I.R. (2011). Decomposition and insect succession of clothed and unclothed carcasses in Western Australia. Forensic Sci. Int..

[44] Hyde E.R., Haarmann D.P., Petrosino J.F., Lynne A.M., Bucheli S.R. (2015). Initial insights into bacterial succession during human decomposition. Int. J. Legal Med..

[45] Roberts L.G., Spencer J.R., Dabbs G.R. (2017). The Effect of Body Mass on Outdoor Adult Human Decomposition. J. Forensic Sci..

[46] Jones A.W., Holmgren A. (2009). Concentration distributions of the drugs most frequently identified in post-mortem femoral blood representing all causes of death. Med. Sci. Law..

[47] Zhou C., Byard R.W. (2011). Factors and processes causing accelerated decomposition in human cadavers—An over view. J. Forensic Leg. Med..

[48] Byrd J.H., Peace M.R., Kobilinsky L. (2012). Entomotoxicology: Drugs, toxins, and insects. Forensic Chemistry Handbook.

[49] Jones A.W., Holmgren A., Ahlner J. (2012). Concentrations of free-morphine in peripheral blood after recent use of heroin in overdose deaths and in apprehended drivers. Forensic Sci. Int..

[50] Abd El-Aziz F.E., Eldeeb S.M., Abdellah N.Z., Shaltout E.S., Ebrahem N.E. (2022). Influence of Scorpion Venom on Decomposition and Arthropod Succession. Egypt. Acad. J. Biol. Sci. B Zool..

[51] Goff M.L. (1993). Estimation of Postmortem Interval Using Arthropod Development and Successional Patterns. Forensic Sci. Rev..

[52] Carvalho L.d., Thyssen P., Goff M., Linhares A. (2004). Observations on the succession patterns of necrophagous insects on a pigcarcass in an urban area of South eastern Brazil. J. Forensic Med. Toxicol..

[53] Martinez E., Duque P., Wolff M. (2007). Succession pattern of carrion-feeding insects in Paramo, Colombia. Forensic Sci. Int..

[54] Gomes L., Gomes G., Desuó I.C. (2009). A preliminary study of insect fauna on pig carcasses located in sugar cane in winter in south eastern Brazil. Med. Vet. Entomol..

[55] Tembe D., Mukaratirwa S. (2021). Insect Succession and Decomposition Pattern on Pig Carrion During Warm and Cold Seasons in Kwazulu-Natal Province of South Africa. J. Med. Entomol..

[56] Thümmel L., Lutz L., Geissenberger J., Pittner S., Heimer J., Amendt J. (2023). Decomposition and insect succession of pig cadavers in tents versus outdoors—A preliminary study. Forensic Sci. Int..

[57] Voss S.C., Spafford H., Dadour I.R. (2009). Annual and seasonal patterns of insect succession on decomposing remains at two locations in Western Australia. Forensic Sci. Int..

[58] Benbow M.E., Pechal J.L., Lang J.M., Erb R., Wallace J.R. (2015). The Potential of High-throughput Metagenomic Sequencing of Aquatic Bacterial Communities to Estimate the Postmortem Submersion Interval. J. Forensic Sci..

[59] Iqbal M.A., Ueland M., Forbes S.L. (2020). Recent advances in the estimation of post-mortem interval in forensic taphonomy. Austr. J. Forensic Sci..

[60] Al-Qahtni A., Mashaly A., Haddadi R., Al-Khalifa M. (2021). Seasonal Impact of Heroin on Rabbit Carcass Decomposition and Insect Succession. J. Med. Entomol..

[61] Al-Khalifa M., Mashaly A., Al-Qahtni A. (2021). Impacts of antemortem ingestion of alcoholic beverages on insect successional patterns. Saudi J. Biol. Sci..

[62] Binh D., Thanh T., Chi P. (2010). Proteomic characterization of the thermostable toxins from Naja naja venom. J. Venom. Anim. Toxins Incl. Trop. Dis..

[63] Pepys M.B., Hirschfield G.M. (2003). C-reactive protein: A critical update. J. Clin. Investig..

[64] Fahim A. (2001). Biochemical effects of crude venoms of two elapidae from Saudi fauna on male albino rats. J. Egypt. Ger. Soc. Zool..

[65] El-Refaei M.F., Sarkar N.H. (2009). Snake venom inhibits the growth of mouse mammary tumor cells in vitro and in vivo. Toxicon.

[66] Evangelista I.L., Martins A.M., Nascimento N.R., Havt A., Evangelista J.S., de Norões T.B., Toyama M.H., Diz-Filho E.B., Toyama Dde O., Fonteles M.C. (2010). Renal and cardiovascular effects of *Bothrops marajoensis* venom and phospholipaseA2. Toxicon.

[67] Schneemann M., Cathomas R., Laidlaw S.T., ElNahas A.M., Theakston R.D., Warrell D.A. (2004). Life-threatening envenoming by the Saharan horned viper (*Cerastes cerastes*) causing micro-angiopathic haemolysis, coagulopathy and acute renal failure: Clinical cases and review. Qjm.

[68] Bazaa A., Marrakchi N., El Ayeb M., Sanz L., Calvete J.J. (2005). Snake venomics: Comparative analysis of the venom proteomes of the Tunisian snakes *Cerastes cerastes*, *Cerastes vipera* and *Macrovipera lebetina*. Proteomics.

[69] Vyas V.K., Brahmbhatt K., Bhatt H., Parmar U. (2013). Therapeutic potential of snake venom in cancer therapy: Current perspectives. Asian Pac. J. Trop. Biomed..

[70] Ozverel C.S., Damm M., Hempel B.F., Göçmen B., Sroka R., Süssmuth R.D., Nalbantsoy A. (2019). Investigating the cytotoxic effects of the venom proteome of two species of the Viperidae family(*Cerastes cerastes* and *Cryptelytrops purpureomaculatus*) from various habitats. Comp. Biochem. Physiol. C Toxicol. Pharmacol..

[71] Mashaly A.M. (2017). Carrion beetles succession in three different habitats in Riyadh, Saudi Arabia. Saudi J. Biol. Sci..

[72] Sonker R., Rawat S., Singh K. (2015). Succession and life cycle of beetles on the exposed carcass. Int. J. Sci. Innov. Res..

[73] Al-Dakhil A.A., Alharbi S.A. (2020). A preliminary investigation of the entomofauna composition of forensically important necrophagous insects in Al-Madinah Al-Munawwarah region, Kingdom of Saudi Arabia. J. Taibah Univ. Sci..

[74] Early M., Goff M.L. (1986). Arthropod succession patterns in exposed carrion on the island of O’ahu, Hawaiian Islands, USA. J. Med. Entomol..

[75] Mabika N., Masendu R., Mawera G. (2014). An initial study of insect succession on decomposing rabbit carrions in Harare, Zimbabwe. Asian Pac. J. Trop. Biomed..

[76] VanLaerhoven S.L., Anderson G.S. (1999). Insect succession on buried carrion in two biogeoclimatic zones of BritishColumbia. J. Forensic Sci..

